# Current status and prospects of traditional Chinese medicine combined with stem cell therapy for chronic kidney disease

**DOI:** 10.3389/fphar.2024.1505206

**Published:** 2025-01-14

**Authors:** Tianyang Qian, Yining He, Chao Han, Ruxue Yan, Weiming He

**Affiliations:** ^1^ Division of Nephrology, Affiliated Hospital of Nanjing University of Chinese Medicine, Jiangsu Province Hospital of Chinese Medicine, Nanjing, China; ^2^ The First School of Clinical Medicine, Nanjing University of Chinese Medicine, Nanjing, China; ^3^ Yancheng Dafeng Hospital of Chinese Medicine, Teaching Hospital of Nanjing University of Chinese Medicine, Yancheng, China

**Keywords:** stem cells, traditional Chinese medicine, renal fibrosis, chronic kidney disease, treatment

## Abstract

Renal fibrosis is one of the main pathological features of chronic kidney disease (CKD), and its treatment has been a hot research topic. Recent studies have shown that stem cell therapy can repair renal pathological changes and slow the progression of CKD. In addition, a large number of experiments have confirmed that traditional Chinese medicine (TCM), especially Chinese medicine compound preparations, has the advantage of multitargeting interventions to improve renal fibrosis. Therefore, stem cell therapy combined with TCM is expected to provide new therapeutic ideas and measures to solve kidney problems. This article reviews the current status of TCM combined with stem cell therapy for CKD, discusses existing problems, and proposes future prospects.

## 1 Introduction

Chronic kidney disease (CKD) is a progressive condition characterized by a gradual loss of kidney function over time. A 2017 Global Burden of CKD study showed that the global prevalence of CKD has exceeded 9%, and CKD patients in China account for 18.97% of the global total (GBD Chronic Kidney Disease Collaboration, 2020). The results of a systematic evaluation and analysis of the prevalence of chronic kidney disease (CKD) in Asia in 2022 showed that the number of CKD patients in China is the highest in Asia, which is facing a significant disease burden (Liyanage et al., 2022). CKD has become a major public health problem in China and worldwide. The most common progression and pathogenesis of CKD to end-stage renal disease (ESRD) is renal fibrosis, which is characterized by the enlargement of the space between the tubular basement membrane and the peritubular capillaries through the deposition of matrix proteins (collagen 1 predominantly) (Humphreys, 2018), which ultimately leads to the impairment of normal renal function. Currently, Western medicine mainly slows the progression of CKD by controlling the causes of renal fibrosis, such as by controlling blood pressure, lowering blood glucose, and regulating immunity. However, these modalities have limited efficacy and the long-term application of these drugs has more adverse effects (Peng et al., 2022). The main treatment for end-stage chronic renal failure (CRF) is renal dialysis or renal transplantation, which are associated with complications, incomplete replacement dialysis, and a scarcity of kidney donors (Peek and Wilson, 2023). In recent years, Chinese medicines and their active ingredients have attracted much attention as potential inducers of stem cell proliferation and differentiation because of their cytokine-like physiological activities (Li et al., 2016). Therefore, emerging stem cell transplantation has brought new hope for the treatment of CKD, and stem cell transplantation combined with Chinese medicine treatment is expected to constitute a new exploration for antifibrosis treatment.

## 2 Mechanism and treatment status of chronic kidney disease fibrosis

Chronic kidney disease (CKD) is defined as structural or functional changes in the kidneys, a glomerular filtration rate (GFR) of less than 60 mL/min/1.73 m^2^, or both for at least 3 months (Webster et al., 2017). CKD, a slowly progressing disease, is characterized by a decline in renal function, tubular injury, oxidative stress, and the inflammatory response (Reiss et al., 2024). During the repair or response process, injured renal tubular epithelial cells and infiltrating inflammatory cells release profibrotic factors, and through a complex signalling cascade of events, myofibroblasts undergo activation, expansion, and deposition of extracellular matrix (ECM) components (Yuan et al., 2019). Fibrosis occurs when the ECM accumulates excessively in the renal parenchyma. Excessive fibrotic deposition leads to changes in the normal renal structure and impedes blood supply, resulting in irreversible renal injury (Panizo et al., 2021; Vanhove et al., 2017). Renal tubular epithelial mesenchymal transition (EMT) is a key step in the development of renal fibrosis and is characterized by an imbalance between myofibroblast proliferation and ECM production and degradation, and preventing renal EMT is essential for slowing the progression of CKD (Zeisberg and Duffield, 2010).

Currently, traditional CKD treatment is still based on controlling the etiology of the disease, such as poorly controlled diabetes mellitus (DM), hypertension, glomerular disease, glomerulonephritis, and autosomal dominant polycystic kidney disease (Webster et al., 2017). The international standard of care for slowing the progression of CKD is to block the renin-angiotensin-aldosterone system (RAAS) with angiotensin-converting enzyme inhibitors, angiotensin II receptor type 1 (AT1) antagonists, or direct renin blockers. In addition to controlling blood pressure, these drugs have been shown to have indirect antifibrotic effects in some experimental studies (Klinkhammer et al., 2017). However, RAAS blockade cannot completely prevent the progression of CKD and may be too late for diagnosis due to the absence of symptoms in the initial phase. When damaged kidneys progress to ESRD, renal replacement therapy, including dialysis or kidney transplantation, can be used. However, dialysis, while removing certain toxic substances from the blood and replacing the filtration function of the kidneys, does not restore other renal functions, such as the production of erythropoietin (EPO) and the activation of vitamin D (National Kidney and Urologic Diseases Information Clearinghouse NKUDIC, 2012). Although transplantation is effective, it is not widely used due to the lack of donor countries (IlicD, 2017). Although transplantation is effective, its widespread use is limited by a shortage of kidney donors, expensive transplantation costs, rejection reactions, and multiple complications resulting from the long-term application of immunosuppressive drugs. Therefore, TCM and stem cell transplantation have attracted much attention as new approaches to slow the progression of CKD.

## 3 Stem cell therapy for renal fibrosis

Stem cells are a class of cells with self-renewal, high proliferation and multidirectional differentiation potential and are classified into embryonic stem cells (ESCs) and adult stem cells (SSCs) according to their developmental stage. ESCs originating from the intraembryonic cell mass or primitive germinal ridge are characterized by self-renewal, clonogenicity and pluripotency, as evidenced by their ability to grow in an undifferentiated state and to differentiate into several cell types of mesodermal, endodermal and ectodermal lineages (IlicD, 2017; Hassani et al., 2019; Evans and Kaufman, 1981). However, ESCs have faced a series of problems in their application, such as ethical issues in the acquisition and treatment of ESCs derived from human blastocysts, uncontrolled growth of ESCs without sufficient differentiation due to unlimited cellular renewal capacity, which is prone to tumor risk, and transplant rejection of ESCs and their derived cells due to immunogenicity (IlicD, 2017). In this context, induced pluripotent stem cells (iPSCs) were developed as another type of cell with pluripotent capacity. iPSCs were originally developed by Takahashi and Yamanaka by reprogramming fibroblasts through the introduction of four genes, namely, Oct3/4, Sox2, c-Myc, and Klf4 (Takahashi and Yamanaka, 2006). iPSCs have fewer complications of immunogenicity and rejection, but still have limitations, including the risk of derivation efficiency and posttransplantation tumor development due to the highly proliferative nature of iPSCs and the risk of insertion region mutations caused by viral vectors integrated into the genome (Lewandowski and Kurpisz, 2016).

**FIGURE 1:**
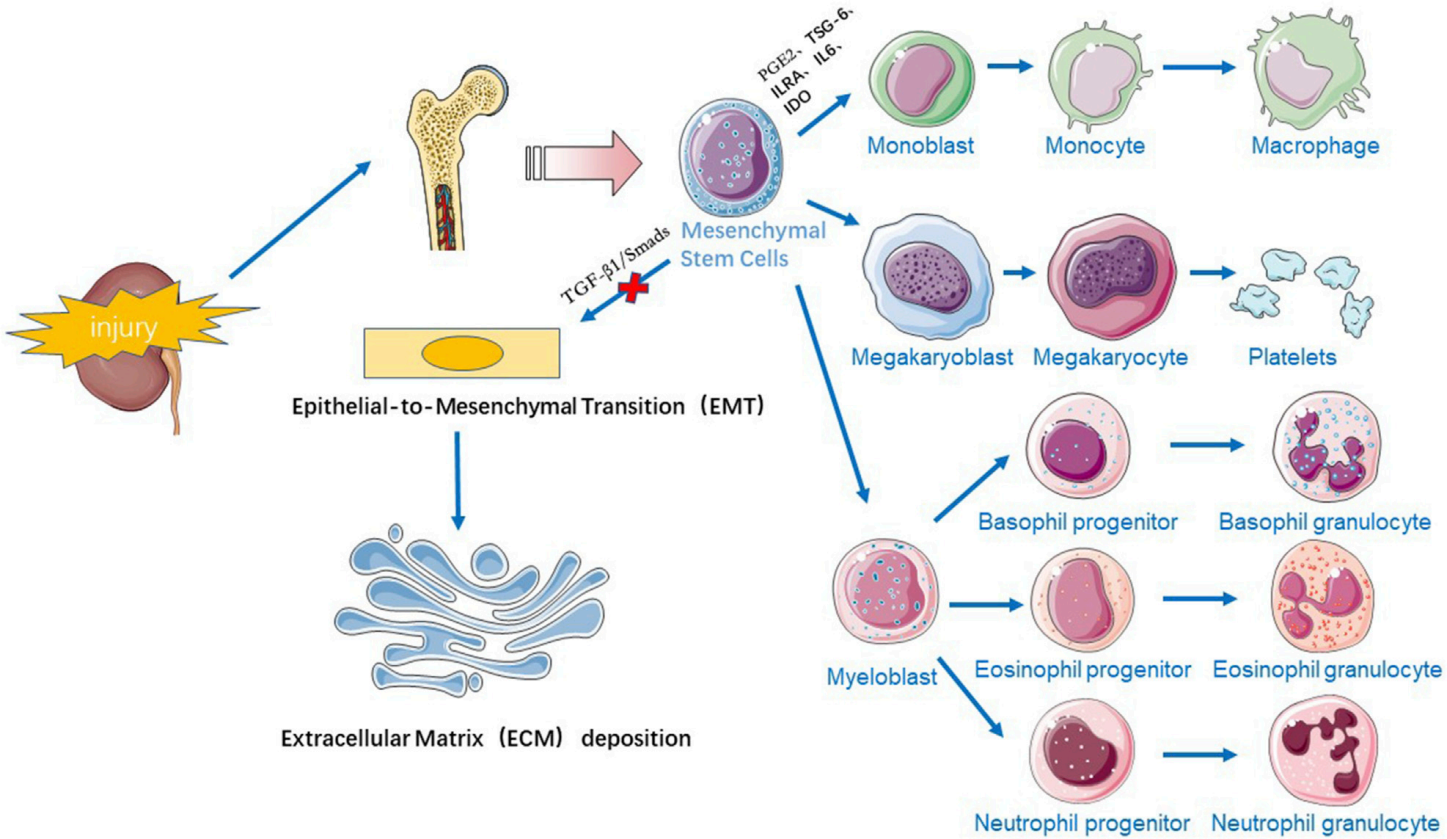
Schematic representation of the mechanisms by which stem cell therapy act on CKD.

Stem cell therapy has emerged as a promising approach for treating CKD due to its potential to regenerate damaged tissues and modulate immune responses. Most of the stem cells used clinically for treating renal fibrosis are SSCs, which have few safety and ethical issues. Mesenchymal stem cells (MSCs), including bone marrow MSCs (BM-MSCs), umbilical cord blood MSCs (UC-MSCs), amniotic fluid MSCs (AF-MSCs), adipose mesenchymal stem cells (AMSCs), Wharton’s jelly mesenchymal stem cells (WJ-MSCs), and dermal mesenchymal stem cells (DMSCs), are among the most effective cell populations for experimental CKD treatment (Papazova et al., 2015; Liu et al., 2021). MSCs can be easily isolated by minimally invasive bone marrow aspiration and liposuction, are cultured *in vitro*, and have been widely used for regeneration of a wide variety of tissues and organs due to their multiple differentiation potential and their ability to migrate, repair, and restore damaged organs (Chung et al., 2015). There are two main mechanisms by which MSCs treat fibrosis (Figure 1).

### 3.1 Anti-inflammatory and immunomodulatory effects

During tissue injury, MSCs located near the bone or originating from the bone marrow, actively “nest” to the site of injury, controlling the inflammatory response (Rustad and Gurtner, 2012) and slowing the process of fibrosis in the damaged kidney. Inflammatory (M1-type) macrophages are proinflammatory and secrete factors such as TGF-β1 and IL-1β to participate in the development of fibrosis, while alternatively activated (M2-type) macrophages secrete IL-10 and arginase-1 to promote inflammation and reduce inflammation (Zhou et al., 2021; Pouyanfard et al., 2021; Wang et al., 2021). MSCs can secrete a variety of factors, such as PGE2, TSG-6, ILRA, IL6, and IDO, which can regulate M1/M2 macrophage function by transmitting mitochondria and other factors. MSCs can regulate the ratio of M1/M2 macrophages, promote macrophage differentiation to the M2 phenotype and inhibit the secretion of proinflammatory factors, thus inhibiting the inflammatory response and promoting tissue repair (Song et al., 2015; Xue et al., 2018). A study revealed that after transplanting MSCs through the tail vein into a unilateral ureteral obstruction (UUO) model, renal interstitial fibrosis was significantly attenuated in the MSC group, and it was confirmed that MSCs could be recruited into injured kidneys and play secretory/paracrine roles, reducing macrophage infiltration and myofibroblast proliferation (Xing et al., 2019). Repeated infusions of UC-MSCs were found to have superior anti-inflammatory effects on other stromal cells, reducing macrophage infiltration and inducing polarization towards the M2 macrophage phenotype (Rota et al., 2018). In addition to regulating the innate immune response, MSCs can also regulate innate and acquired immunity. MSCs can block T cells and B cells in the G0/G1 phase of the cell cycle and can play an immunomodulatory role by inducing the recruitment and production of regulatory T cells (Tregs) (Zhou et al., 2018). The IL4Rα/JAK3/STAT6 signaling pathway plays an important role in the activation of myeloid fibroblasts, the polarization of M2 macrophages and the development of renal fibrosis. Signal transducer and activator of transcription 6 (STAT6) is phosphorylated in the kidney in response to obstructive injury or folate injury, driving macrophage polarization to the profibrotic M2 phenotype and increasing renal ECM proteogenesis and collagen deposition. Specific inhibitors of STAT6 reduce M2 macrophage polarization and decrease the M2 macrophage markers Arg1, MRC1, Fizz1, and CCL17, preventing renal dysfunction. STAT6 deficiency significantly reduces the number of M2 macrophage phenotypic marker PDGFR-β and CD45 double-positive cells, preventing M2 macrophage polarization and monocyte-to-fibroblast transition in folate nephropathy (Jiao et al., 2021a). In angiotensin II (Ang II)-induced hypertensive nephropathy, myeloid phosphatase and tensin homologue (PTEN) knockout mice presented a significant increase in the accumulation of double-positive cells for CD45 and PDGFR-β, which increased the infiltration of macrophages and T lymphocytes into the kidney and exacerbated renal injury and fibrosis in Ang II-induced hypertension (Jiao et al., 2021b). In addition to the STAT6 and PTEN genes, Jumonji structural domain-containing protein-3 (JMJD3) affects renal fibrosis. When renal obstructive injury occurs, JMJD3 is induced and leads to H3K27 demethylation, M2 macrophage polarization, and the development of renal fibrosis. Changlong An et al. reported that in JMJD3-deficient mice, CD206 expression is significantly reduced, and the expression of IFN regulatory factor 4 (IRF4), a target gene of JMJD3, is also decreased (An et al., 2023).

### 3.2 Inhibition of EMT

EMT plays an important role in the pathogenesis of renal fibrosis, and MSCs can inhibit fibrotic signalling pathways to alleviate renal fibrosis. Transforming growth factor β1 (TGF-β1), a fibrosis-inducing factor, accelerates tissue fibrosis by regulating EMT, and Smads, a downstream target molecule of TGF-β1, induces an increase in the production of ECM after phosphorylation (p-Smads) induced by TGF-β1 (Bai et al., 2024). The TGF-β1/Smad signalling pathway plays a key role in the process of kidney fibrosis. Xiang et al. (2020) reported that UC-MSCs significantly reduced TGF-β levels and downregulated the tubular expression of fibroblast markers (e.g., α-SMA and type IV collagen) in rats with diabetic nephropathy (DN), which effectively improved renal function and inhibited inflammation and fibrosis. Yu et al. (2019) showed that UC-MSCs inhibited the expression of TGF-β1 and p-Smad2/3, improved renal morphology, and reduced the deposition of collagen fibres in a mouse model of aristolochic acid (AA)-induced renal fibrosis induced by tail-vein injection, confirming the ability of UC-MSCs to attenuate EMT through the inhibition of the TGF-β/Smad signalling pathway. In addition to the TGF-β/Smad pathway, the NF-κB, MAPK/ERK, PI3K/AKT and TNF-α signalling pathways also alleviate renal fibrosis (Liu et al., 2021). Yu et al. (2022) also found experimentally that MSCs could inhibit EMT by inhibiting the galectin-3/Akt/GSK-3β/snail signalling pathway, thus ameliorating renal interstitial fibrosis.

## 4 Combined treatment of renal fibrosis with traditional Chinese medicine

Traditional Chinese medicine (TCM) has a long history of treating kidney diseases, with a focus on restoring balance and harmony within the body. At present, experiments have shown that natural products in Chinese herbs such as flavonoids, terpenoids, polyphenols, alkaloids, quinones, and glycosides, perform positively in the process of treating renal fibrosis due to their antioxidant and anti-inflammatory pharmacological effects, and some natural products or Chinese medicines can be used to regulate Wnt/beta-catenin, MAPK, SIRT1/PGC-1α, PI3K/Akt, AMPK/mTOR, JAK2/STAT3, TGF -β/Smad and other signalling pathways, reducing transforming growth factor levels, reversing Nrf2 levels and fibronectin expression, and other pathways to inhibit renal fibrosis (Zhou et al., 2022; Peng et al., 2022). The use of traditional Chinese medicine (TCM) for the treatment of renal fibrosis has gradually increased, and based on basic TCM theories on stem cells, several traditional Chinese medicines have been found to enhance stem cell function, and these findings provide a new method for renal fibrosis treatment.

### 4.1 Traditional Chinese medicine theory on stem cells

#### 4.1.1 The concept of stem cells

Currently, the understanding of stem cells in Chinese medicine primarily starts from homogeneity with “kidney essence.” From the perspective of stem cell formation, embryonic stem cells are derived from the internal cell mass of the embryo at the blastocyst stage, starting from the formation of fertilized egg to the formation of mulberry embryo through multiple rounds of cleavage, mulberry embryo through the cleavage of the sphere of polarization and rearrangement of cavitation and ultimately the formation of blastocysts consisting of trophoblastic ectoderm and the internal cell mass (Yang et al., 2024), the “Spiritual Pivot - Division of Qi” said: “When a man and a woman copulate, their essences, namely, the sperm and ovum, interact to generate a brand new essence (Jing) of another human being which then develops into the physical figure of an infant.” (Chen, 2016) The innate essence of kidney essence is endowed by the parents and is the original material that constitutes the embryo; therefore, the stem cells that originate from the fertilized egg and are born before the body are the same as the “innate essence” in terms of origin.

#### 4.1.2 Physiological functions of stem cells

**FIGURE 2:**
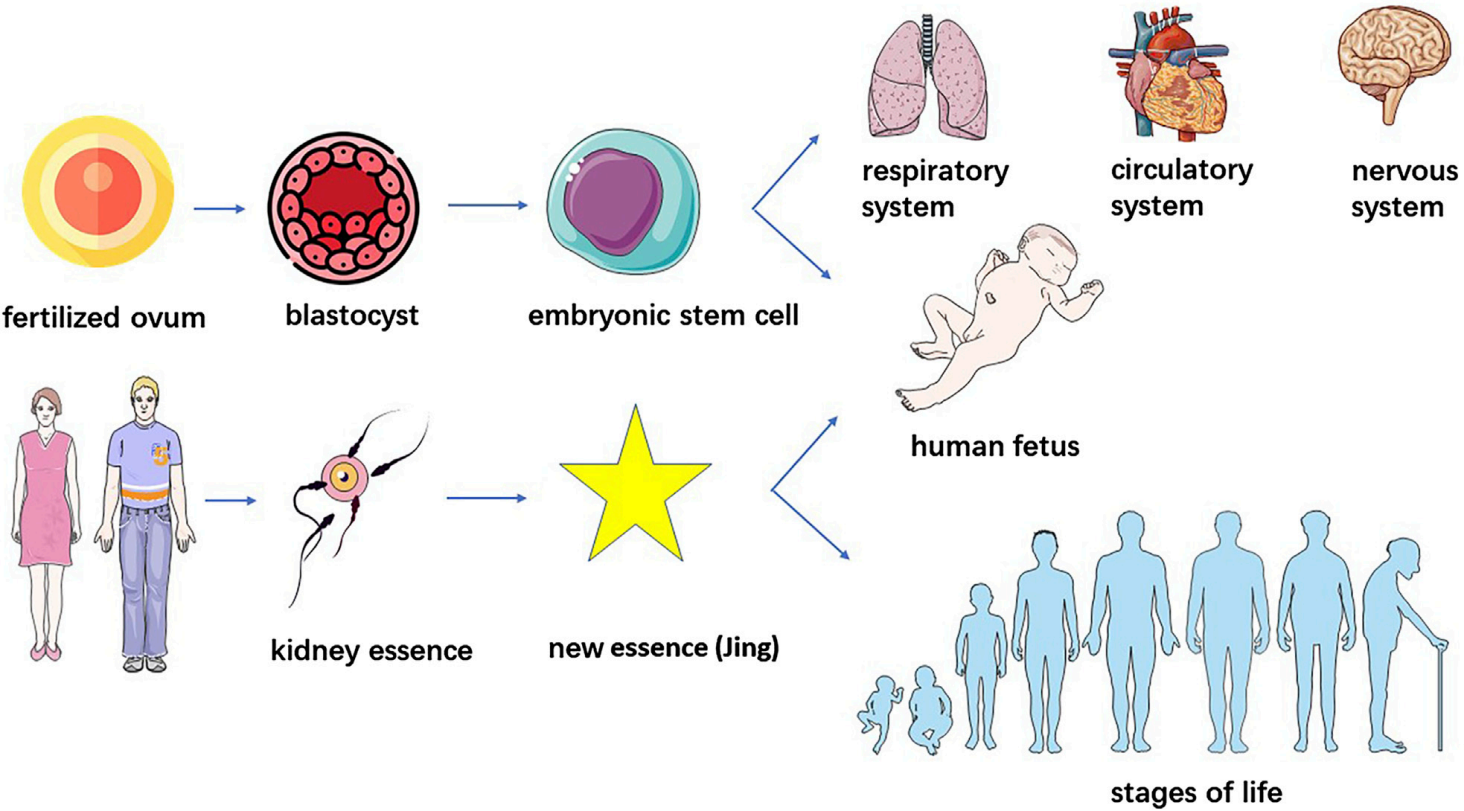
Identity of stem cells with “kidney essence.”

Stem cells, are a class of multipotential cells with self-replication and self-division abilities that are capable of regenerating various tissues, organs and the human body, and when stem cells age due to oxidative stress and other reasons, these cells deteriorate tissue renewal and regeneration (Zhu and Luo, 2022). SSCs can maintain various physiological functions of organs and tissues through self-renewal, multidirectional or unidirectional differentiation and damage, repair and can differentiate into various types of functional cells such as pancreatic islet cells, haematopoietic cells, cardiomyocytes, neuronal cells, and hepatocytes, which are widely distributed in tissues and organs to promote the development and maturation of various tissues and organs, and participate in their functional activities. Stem cells from the bone marrow can be transported throughout the body with the bloodstream and differentiate into new cardiomyocytes, neuronal cells, liver cells, etc., in damaged tissues to perform repair and regeneration (Hui et al., 2022; Wanting et al., 2024). On the other hand, the senescence of stem cells results in the loss of lineage specificity and the production of nonfunctional differentiation products, leading to loss of tissue integrity and a decline in physiological function. With the aging of MSCs, an increase in the expression of proinflammatory cytokines not only weakens the proliferation of cells, but also inhibits the osteogenic differentiation of MSCs, resulting in an imbalance in bone metabolism, which induces osteoporosis. Huangdi Neijing Suwen – The Treatise on Ancient Theory of True Qi states, “The kidney qi of an 8 year old boy begins to develop. His hair grows long, his teeth change. When a man reaches the age of sixteen, his kidney qi is fully developed. Tian gui arrives, bringing with it the fullness of essential qi and secretion of sperm. When yin and yang are harmonized at this time, a child is conceived. When he is 24 years old, his kidney qi permeates normally, his wisdom teeth appear and he has reached his full height. At the age of thirty-two, he is muscular with strong bones and tendons. At the age of forty, his kidney qi begins to decline, with his hair falling off and teeth withering. When he reaches the age of forty-eight, yang qi decays at the upper part, his face loses luster and hair turns grey. At the age of fifty-six, liver qi declines, movements of tendons are hampered. At the age of sixty-four, tian gui is exhausted, essence is reduced and health fails, hair and teeth fall. The kidney governs the water and receives and stores the essence from the five viscera and six bowels. Only when the five viscera are strong in action, could the kidney be able to emanate. But now the five viscera are exhausted, with debilitated tendons and bones. Tian gui is totally exhausted. Thus his hair turns white, his body becomes heavy, his walking is unsteady and is unable to produce offspring.” (Li, 2011) Kidney essence determines growth, development and strength on the one hand, and influences the aging process on the other hand (Zhu, 2005). Therefore, stem cells and kidney essence are also homogeneous in terms of physiological functions and aging mechanisms (Figure 2). Some scholars believe that the function of stem cells is also in line with the law of the five elements. The differentiation process of stem cells seems to involve five elements, and the ability of a certain number of stem cells to prevent them from becoming overgrowth seems to involve these five elements (Wen et al., 2017).

### 4.2 Effect of traditional Chinese medicine on stem cells

Based on the above understanding of stem cells, some studies have shown that kidney tonic prescription drugs can promote the proliferation, homing and differentiation of SSCs, and delay aging. Li et al. (2017) reported that icariin (ICA) could enhance the pluripotency of human umbilical cord mesenchymal stem cells (huMSCs), and ICA-treated huMSCs reduced the level of fibrosis in CRF rats on the 14th day post transplantation. Infused ICA-treated huMSCs preferentially migrated to injured renal tissues, promoted cell regeneration and growth factor secretion, inhibited oxidative damage and the inflammatory response, and improved renal function. Xu et al. (2017) experimentally confirmed that the main components of Epimedium flavonoids promote the osteogenic differentiation of BMSCs and that this effect is mainly achieved by activating 8 related signalling pathways, such as the oestrogen signalling pathway, p38 in the MAPK signalling pathway and 33 proliferation and differentiation related targets. Gao et al. (2020) reported that Astragaloside IV (AS-IV), one of the main active ingredients of Astragalus, combined with BMSCs could reduce the levels of α-smooth muscle actin (α-SMA) and desmin in diabetic nephropathic rats, improve renal pedicle cell transdifferentiation, and alleviate nephropathy and desmin, improve renal podocyte transdifferentiation, and alleviate renal pathological damage. It has also been found that ganoderic acid-D, the main active ingredient of Ganoderma lucidum, can inhibit D-galactose-induced senescence of senescent human amniotic MSCs and promote stem cell proliferation by increasing the expression of 14-3-3ε protein in the 14-3-3 family of proteins, and by activating the calmodulin/calmodulin type 2 kinase/Nrf2 signalling axis (Xu et al., 2020; Yuan et al., 2022). In addition to single prescriptions of traditional Chinese medicine, some compound preparations also have the same effect. Liu et al. (2015) used the formula Tonifying Kidney and Activating Blood Tang selected from Zhao Lian’s Dacheng of Injuries of the Qing Dynasty and revealed that Tonifying Kidney and Activating Blood Tang containing medicinal serum could promote the proliferation of rat BMSCs. Gao et al. (2014) demonstrated that an effective combination of four Chinese medicines, Epimedium, Psoralea, Ligustrum and Polygonum multiflorum, could upregulate the gene expression of Smad4 and promote the proliferation of BM-MSCs. Ding et al. (2017) selected the decoction of Zuo Gui Wan for *in vitro* intervention on MSCs and observed that Zuo Gui Wan could improve the morphology and structure of senescent MSCs, and promote their proliferative ability. Wang (2016) confirmed that renal fibrosis was significantly reduced in FSGS model rats treated with Zuo Gui Wan combined with stem cell transplantation, and that BMSCs significantly inhibited renal fibrosis and protected renal function by inhibiting the TGF-β1/Smad3 pathway and reducing the expression of plasminogen activator inhibitor 1 (PAI1) (Fan et al., 2020). Many *in vitro* experimental studies have shown that renal tonic formulae can promote the proliferation and antiapoptosis effects of BMSCs, enhance the secretion function of stem cells, and affect the local microenvironment to promote the survival and function of BMSCs. Chinese medicinal molecules and their extracts are relatively easy to obtain and play important roles in solving the problems of endogenous stem cell deficiency, stem cell directed differentiation and functional regulation (Zhang et al., 2024).

## 5 Challenges and future prospects

As a major pathological manifestation of chronic kidney disease with high morbidity and mortality, renal fibrosis has always been a target for treatment. TCM has great potential for treatment by guiding the proliferation, homing and differentiation of stem cells, but current studies are mainly based on animal and cellular experiments and lack clinical application, and additional mechanisms of action have yet to be confirmed for the sake of safety and efficacy. Efficient delivery and homing of stem cells to the injured kidney remain significant challenges, and variability in herbal composition and quality control poses challenges for consistent therapeutic outcomes. Combining TCM with advanced biomedical techniques, such as gene editing and biomaterials, may further enhance therapeutic outcomes. In addition, most of the understanding of stem cells under the guidance of Chinese medicine theory starts from kidney essence. Whether there are more herbs that can help protect stem cells against fibrosis from the perspective of yin and yang, five elements and meridian theory is also worth exploring, and we hope that the reflections in this paper can provide help for further research in the future.

## 6 Conclusion

The combination of traditional Chinese medicine and stem cell therapy, aimed at enhancing the efficacy of both treatments, offers a promising strategy for treating chronic kidney disease. TCM can not only improve the microenvironment of the kidneys, making it more conducive to stem cell survival and differentiation but also, regulate immune responses and reduce inflammation, enhancing the regenerative capacity of stem cells. While challenges remain, ongoing research and advancements in both fields hold potential for improving patient outcomes. Future studies should focus on optimizing treatment protocols and conducting rigorous clinical trials to validate the therapeutic benefits of this integrated approach.
